# Supplementary material for: A Refractive Index-Based Dual-Band Metamaterial Sensor Design and Analysis for Biomedical Sensing Applications
Source: Sensors (Basel). 2025 Jan 3;25(1):232. doi: 10.3390/s25010232 (PMC11723037; doi:10.3390/s25010232)
Supplement: Supplementary file 1 [file sensors-25-00232-s001.zip › sensors-3261904-supplementary.pdf]

Supplementary information

A Refractive Index-Based Dual Band Metamaterial Sensor Design and Analysis for Biomedical Sensing Applications

Lakshmi Darsi 1, †, ‡, and Goutam. Rana 1, †, ‡ *

1 Department of ECE, SRM University AP; lakshmi_darsi@srmap.edu.in, goutam.r@srmap.edu.in

* Correspondence: goutam.r@srmap.edu.in † SRM University, Guntur, Andhra Pradesh, India.

‡ These authors contributed equally to this work.

---

1. Calculation methodology of quality (Q)-factor and sensitivity (S)
   - I) Water and glucose detection
   - II) HIV and M13 viruses
   - III) Breast, skin, MCF-7 and PC12 cancers

The quality factor (Q) and sensitivity (S) of a sensor is calculated by using the following formulas.

$Q = \frac{f_r}{FWHM}$, where f_r is resonant frequency, FWHM is full width half maxima and Q is quality factor.

$S = \frac{\nabla f}{\nabla n}$, where ∇f is change in resonant frequency, ∇n is change in refractive index and s is the sensitivity.

I) Water and Glucose detection:

Without analyte (free space):

Refractive index for free space, n=1

Resonance frequencies of proposed sensor, fr1=0.75THz and fr2=1.01THz

With analyte (water):

$fr_1 = 0.732$

FWHM1 = 0.75-0.7165 = 0.034

$\Delta fr_1 = 0.75\text{-}0.732 = 0.018$

$\Delta n = 1.3198\text{-}1 = 0.3198$

$S_1 = \frac{\Delta fr_1}{\Delta n} = \frac{0.018}{0.3198}$

S_1=0.05628 THz

$fr_2 = 0.987$

FWHM2 = 1.017-0.9615 = 0.055

$\Delta fr_2 = 1.01\text{-}0.987 = 0.023$

$\Delta n = 1.3198\text{-}1 = 0.3198$

$S_2 = \frac{\Delta fr_2}{\Delta n} = \frac{0.023}{0.3198}$

S_2=0.072 THz

$S_1 \approx 56.28$ GHz

$S_2 \approx 72$ GHz

$Q_1 = \frac{0.732}{0.034}$ =21.53

$Q_2 = \frac{0.987}{0.055} = 17.94$

$FOM_1 = \frac{0.5628}{0.034} = 1.6$

$FOM_2 = \frac{0.072}{0.055} = 1.3$

With analyte (Glucose):

$fr_1 = 0.7305$

$fr_2 = 0.9855$

FWHM1 = 0.748-0.714 = 0.034

FWHM2 = 1.0155-0.9585 = 0.057

$\Delta fr_1 = 0.75-0.7305 = 0.0195$

$\Delta fr_2 = 1.01-0.9855 = 0.0245$

$\Delta n = 1.3594-1.3198 = 0.0396$

$\Delta n = 1.3594-1.3198 = 0.0396$

$S_1 = \frac{\Delta fr_1}{\Delta n} = \frac{0.0195}{0.0396}$

$S_2 = \frac{\Delta fr_2}{\Delta n} = \frac{0.0245}{0.0396}$

S_1 =0.49242 THz

S_2 =0.61868 THz

$S_1 \approx 492.42$ GHz

$S_2 \approx 618.68$ GHz

$FOM_1 = \frac{0.49242}{0.034} = 14.48$

$FOM_2 = \frac{0.61868}{0.057} = 10.85$

$Q_1 = \frac{0.7305}{0.034} = 21.48$

$Q_2 = \frac{0.9855}{0.057} = 17.23$

II) HIV and M13:

Without analyte (free space):

Refractive index for free space, n=1

Resonance frequencies of proposed sensor, fr1=0.75THz and fr2=1.01THz

With analyte (HIV):

Refractive index (n) of healthy red blood cell (HRB) and HIV, n_{HRB}=1.399 and n_{HIV}=1.5.

$fr_1 = 0.7215$

$fr_2 = 0.975$

FWHM1 = 0.7155-0.7395=0.034

FWHM2 = 1.005-0.948=0.057

$\Delta fr_1 = 0.75-0.7215 = 0.0285$

$\Delta fr_2 = 1.01-0.975=0.035$

$\Delta n = 1.5-1.399=0.101$

$\Delta n = 1.5-1.399=0.101$

$S_1 = \frac{\Delta fr_1}{\Delta n} = \frac{0.0285}{0.101}$

$S_2 = \frac{\Delta fr_2}{\Delta n} = \frac{0.035}{0.101}$

S_1=0.28217 THz

S_2=0.34653 THz

$S_1 \approx 282.17$ GHz

$S_2 \approx 346.53$ GHz

$Q_1 = \frac{0.7215}{0.034}$ =21.22

$Q_2 = \frac{0.975}{0.057} = 17.10$

$FOM_1 = \frac{0.0.28217}{0.034} = 8.3$ $FOM_2 = \frac{0.34653}{0.057} = 6.08$

With analyte (M13):

Refractive index (n) of healthy red blood cell (HRB) and M13, n_{HRB}=1.399 and n_{M13}=1.57.

$fr_1 = 0.717$ $fr_2 = 0.97$

FWHM1 = 0.735-0.702 = 0.033 FWHM2 = 1.0005-0.9435=0.057

$\Delta fr_1 = 0.75\text{-}0.717\text{=}0.033$ $\Delta fr_2 = 1.01\text{-}0.97\text{=}0.04$

$\Delta n = 1.57\text{-}1.399\text{=}0.171$ $\Delta n = 1.57\text{-}1.399\text{=}0.171$

$S_1 = \frac{\Delta fr_1}{\Delta n} = \frac{0.033}{0.171}$ $S_2 = \frac{\Delta fr_2}{\Delta n} = \frac{0.04}{0.171}$

S_1 =0.193 THz S_2 =0.23392THz

$S_1 \approx$ 193 GHz $S_2 \approx$ 233.92GHz

$FOM_1 = \frac{0.193}{0.033} = 6.4$ $FOM_2 = \frac{0.23392}{0.171} = 4.1$

$Q_1 = \frac{0.717}{0.033} = 21.72$ $Q_2 = \frac{0.97}{0.171} = 17.01$

III) Breast, skin, MCF-7 and PC12:

Without analyte (free space):

Refractive index for free space, n=1

Resonance frequencies of proposed sensor, fr1=0.75THz and fr2=1.01THz

With analyte (Healthy breast cell):

Refractive index (n) of healthy breast healthy cell (HBC) and cancer breast cell (CBC), n_{HBC}=1.449 and n_{CBC}=1.5811.

$fr_1 = 0.726$ $fr_2 = 0.981$

FWHM1 = 0.03 FWHM2 = 0.057

$\Delta fr_1 = 0.024$ $\Delta fr_2 = 0.029$

$Q_1 = \frac{0.726}{0.03}$ =24.20 $Q_2 = \frac{0.981}{0.057} = 17.20$

With analyte (Cancer breast cell):

$fr_1 = 0.717$ $fr_2 = 0.97$

FWHM1 = 0.033 FWHM2 = 0.057

$\Delta fr_1 = 0.75\text{-}0.717\text{=}0.033$ $\Delta fr_2 = 0.041$

$\Delta n = 1.5811\text{-}1.449\text{=}0.1321$

$S_1 = \frac{\Delta fr_1}{\Delta n} = \frac{0.033}{0.1321}$

$S_1 = 0.2498THz$

$S_1 \approx 250$ GHz

$FOM_1 = \frac{0.2498}{0.03} = 8.3$

$Q_1 = \frac{0.717}{0.03} = 23.90$

$\Delta n = 1.5811\text{-}1.449\text{=}0.1321$

$S_2 = \frac{\Delta fr_2}{\Delta n} = \frac{0.041}{0.1321}$

$S_2 = 0.31037THz$

$S_2 \approx 310.37$ GHz

$FOM_2 = \frac{0.31037}{0.057} = 5.44$

$Q_2 = \frac{0.969}{0.057} = 17$

Note: Calculation methodology of quality (Q)-factor and sensitivity (S) as demonstrated for breast cancer detection, and same methodology is used for analyzing skin, MCF-7 and PC12 cancers.
